# Comparison of short-term and long-term clinical effects of modified overlap anastomosis and conventional incision-assisted anastomosis in laparoscopic total gastrectomy

**DOI:** 10.1186/s12893-023-02212-2

**Published:** 2023-10-10

**Authors:** Chu-Ying Wu, Jian-An Lin, Qiao-Zhen Huang, Jian-Hua Xu, Wen-Jin Zhong, Wen-Gui Kang, Jin-Tian Wang, Jun-Xing Chen, Hui-Da Zheng, Kai Ye

**Affiliations:** Department of Gastrointestinal Surgery, The Second Affiliated Hospital of Fujian Medical University, Quanzhou, 362000 China

**Keywords:** Gastric cancer, Gastrointestinal tract, Reconstructive surgical procedures, Surgical staplers, Laparoscopy

## Abstract

**Background:**

To compare short-term and long-term clinical effects of modified overlap anastomosis and conventional incision-assisted anastomosis for laparoscopic total gastrectomy.

**Methods:**

This retrospective cohort study included patients with gastric cancer admitted to the Second Affiliated Hospital of Fujian Medical University from January 2016 to March 2020. Quality of life, intraoperative and postoperative conditions were analyzed.

**Results:**

Compared with the conventional assisted group, the modified overlap group showed a shorter auxiliary incision, milder postoperative pain, shorter time to the first postoperative anal exhaust, shorter time to the first postoperative liquid food intake, and shorter postoperative stay. There were no differences between the two groups regarding operation time, esophagus-jejunum anastomosis time, intraoperative blood loss, number of lymph nodes dissected, and length of the upper incision margin. There were no differences between the two groups regarding postoperative early and late complications. There were no differences between the two groups regarding the QLQ-C30 scale three years after the operation. The scores of the QLQ-STO22 scale 3 years after the operation showed significantly lower scores for dysphagia and feeding limit in the modified overlap group than those in the conventional assisted anastomosis group. There was no recurrence in the modified overlap group but one patient in the conventional assisted group.

**Conclusions:**

Patients undergoing totally laparoscopic total gastrectomy with modified overlap anastomosis have better minimal invasiveness and faster post-operative recovery than conventional incision-assisted anastomosis.

## Introduction

Gastric cancer encompasses the tumors of the stomach, including tumors of the noncardia and the subcardia (Siewert type III), with a center starting 2–5 cm below the esophagogastric junction (EGJ) [1, 2]. The number of new gastric cancer cases was estimated at 1,033,701 in 2018 worldwide, with 782,685 deaths [3]. Gastric cancer incidence is highest in Eastern Asia, Eastern Europe, and South America [1, 4]. More men are affected than women [1]. The direct cause of gastric cancer is not clear, but *Helicobacter pylori* infection and some hereditary cancer predisposition syndromes may play a role [1, 2, 4]. Patients often present with nonspecific symptoms that may include anorexia, weight loss, abdominal pain, dyspepsia, vomiting, and early satiety [1, 4]. The management of gastric cancer is multidisciplinary, but tumor resection or gastrectomy plays a central role [2, 4].

First reported by Kitano et al. in 1994 [5], laparoscopic surgery for gastric cancer is widely applied in clinical practice [6]. Early laparoscopic surgery is mainly used for distal gastric cancer with confirmed safety and effectiveness [7, 8]. Recently, as the incidence of proximal gastric cancer increased, the research focus of laparoscopic surgery for gastric cancer has shifted to total gastrectomy [6, 9]. The incidence of anastomotic complications in laparoscopy-assisted surgery is higher than that in open surgery [10].

Linear and circular staplers are key tools for reconstructing the digestive tract in laparoscopic surgery for gastric cancer [10–13]. The use of a linear stapler for anastomosis with a totally laparoscopic approach is highly valued for its advantages, including a good surgical field of vision, small trauma, and no limitation of patient size [14, 15]. The conventional laparoscopic incision-assisted anastomosis is mainly performed using a circular stapler outside the abdominal cavity through an incision to complete the esophagojejunostomy. In 2010, Inaba et al. [16] proposed the totally laparoscopic overlap method, which is completely performed using linear staplers inside the abdominal cavity to complete the esophagojejunostomy. This method has better vision and minor trauma and is not limited by the patient’s body size [16].

At the authors’ hospital, the overlap method was modified by involving the rotation of the esophagus and modifying the surgeons’ position, the method of esophagojejunostomy, and the closure of the anastomosis. Hence, this retrospective study aimed to compare the postoperative characteristics and anastomosis complications of the modified overlap anastomosis and conventional incision-assisted anastomosis for laparoscopic total gastrectomy.

## Methods

### Study subjects

This retrospective cohort study included patients with gastric cancer admitted to the Second Affiliated Hospital of Fujian Medical University from January 2016 to March 2020 and divided into two groups depend on receiving the method of Gastrectomy. This study followed the principles of the Declaration of Helsinki. The present study was approved by the Second Affiliated Hospital of Fujian Medical University (approval number: 2020330). Written informed consent was obtained from the patients.

### Inclusion and exclusion criteria

The inclusion criteria were 1) tumor located in the middle and upper stomach or the stomach’s fundus, 2) preoperative imaging showed no swollen lymph nodes fused into clusters or distant metastasis, and 3) no severe organ dysfunction and underwent surgery. The exclusion criteria were 1) tumor invading the abdominal cavity of the esophagus for > 2 cm, 2) distant metastasis, or 3) intraoperative conversion to laparotomy.

### Operation methods

#### Gastrectomy

All patients received static inhalation combined with general anesthesia. The patient was placed supine with the legs apart and the head slightly higher. The surgeon stood on the left side of the patient, the assistant stood on the right side, and the laparoscope operator stood between the patient’s legs. Using the 5-hole method, the liver was routinely suspended to free the surgical field. The scope of lymph node dissection followed the 2014 Japanese Gastric Cancer Treatment Guidelines [17]. The Roux-en-Y method was used for esophagus and jejunum reconstruction. After completing the D2 lymph node dissection and total gastric freeing, the front of the diaphragmatic esophageal hiatus and the left diaphragmatic foot were opened to free the lower esophagus fully. A linear stapler was used to cut the duodenum to prepare for digestive tract reconstruction. After separating the esophagus with a linear stapler, the excised whole stomach specimen was put into the specimen bag, which was taken out through a small incision in the upper abdomen area or 3–5 cm around the umbilicus.

#### Digestive tract reconstruction with the modified overlap method

**Fig. 1:**
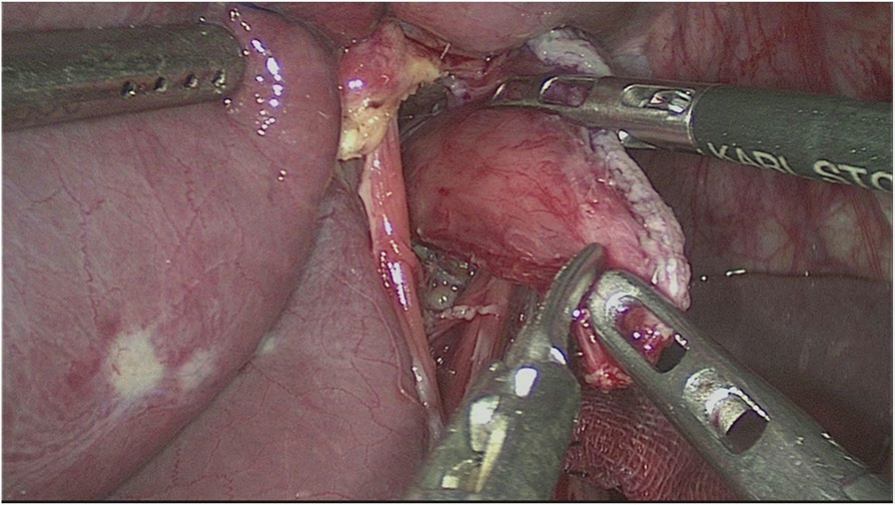
The esophagus was rotated 90° clockwise to make its cutting and closure lines in a sagittal position

**Fig. 2:**
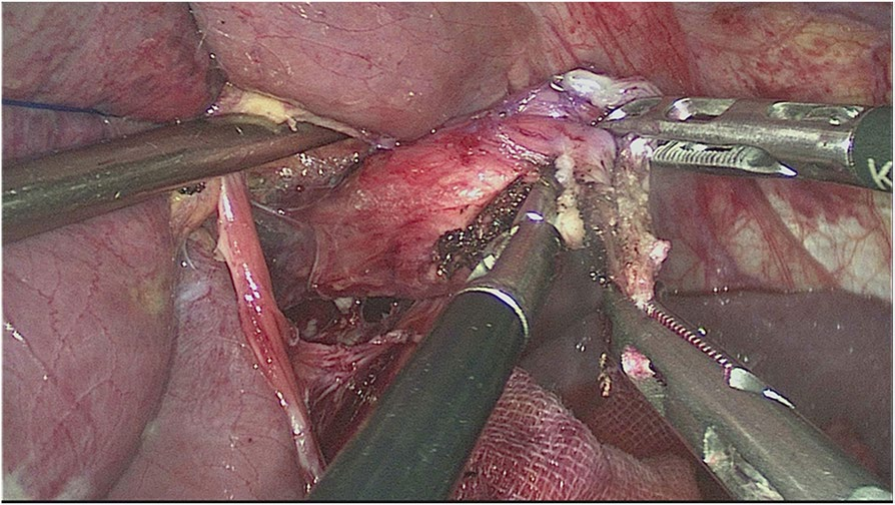
An ultrasonic knife or electric spatula was used to cut the closure line parallel to the esophagus on the right posterior wall of the esophagus’s broken end from the dorsal side to the ventral side to expose the mucosa

**Fig. 3:**
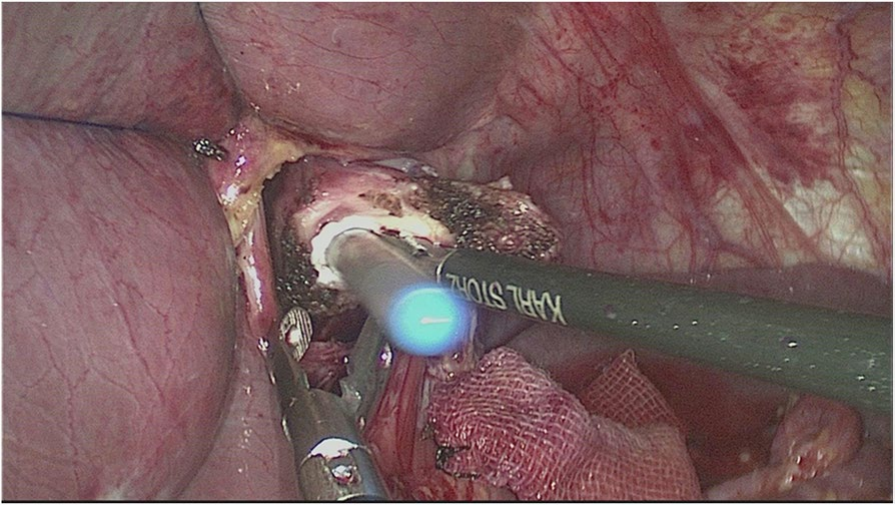
An intraoperative gastric tube was used to guide the anastomotic channel

**Fig. 4:**
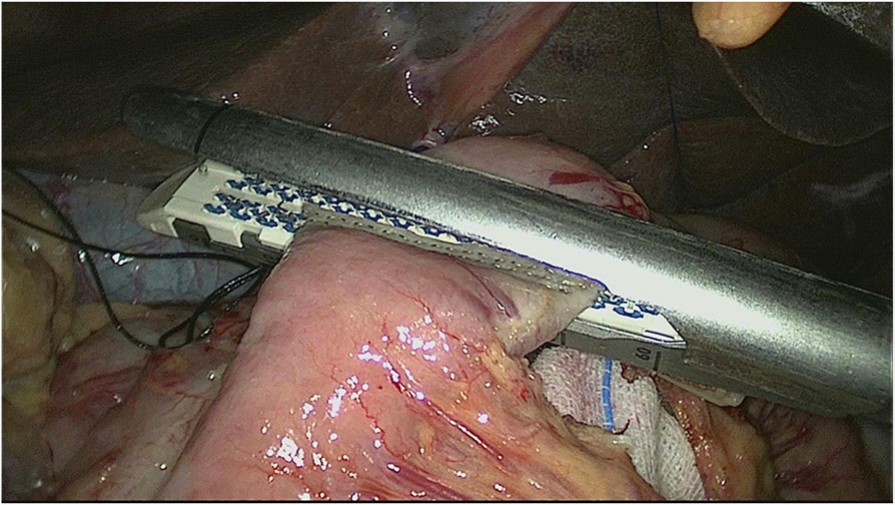
The jejunum and its mesangium were cut 20 cm from the ligament of flexion

**Fig. 5:**
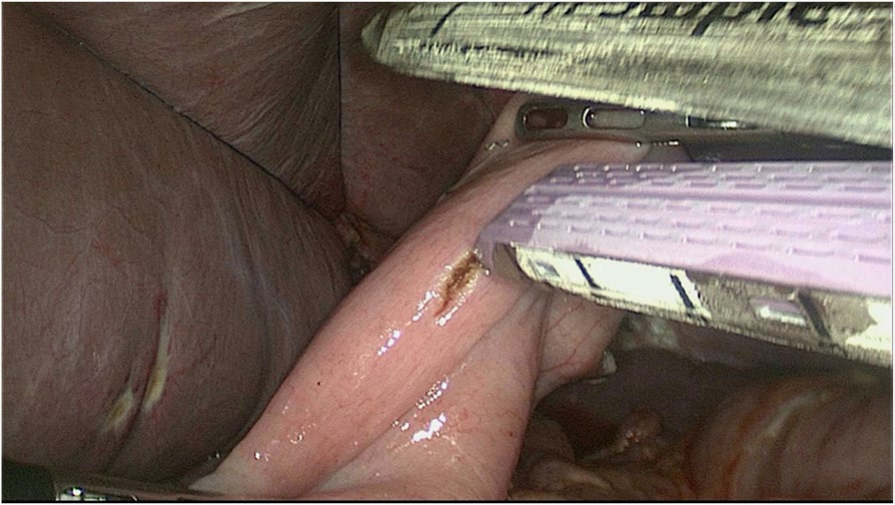
A small hole was made at the contralateral mesangial border 5 cm from the jejunum’s distal end, and a 45-mm linear stapler was inserted via the 12-mm trocar on the upper left side

**Fig. 6:**
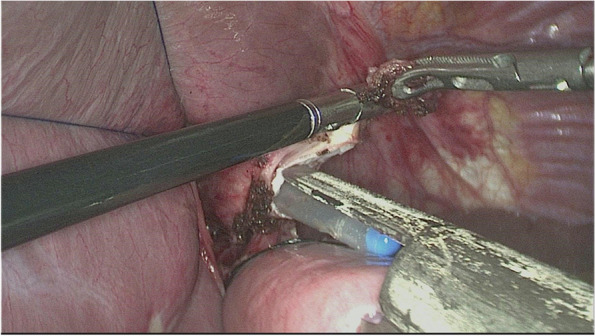
The arms of the linear stapler were put into the opening of the esophagus and the jejunum. It was inserted to the stapling surface through the jejunum opening and to the non-stapling surface through the esophagus opening, guided by the stomach tube

**Fig. 7:**
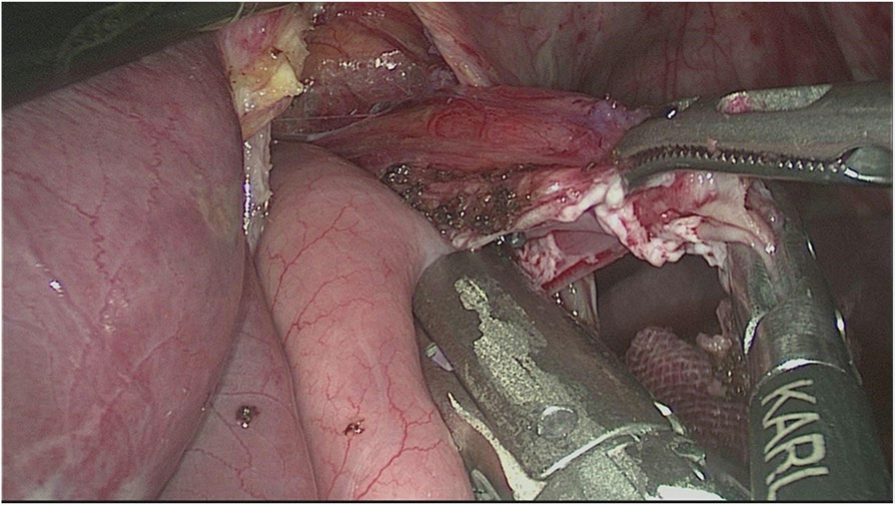
The esophagus-jejunum side-to-side anastomosis was performed

**Fig. 8:**
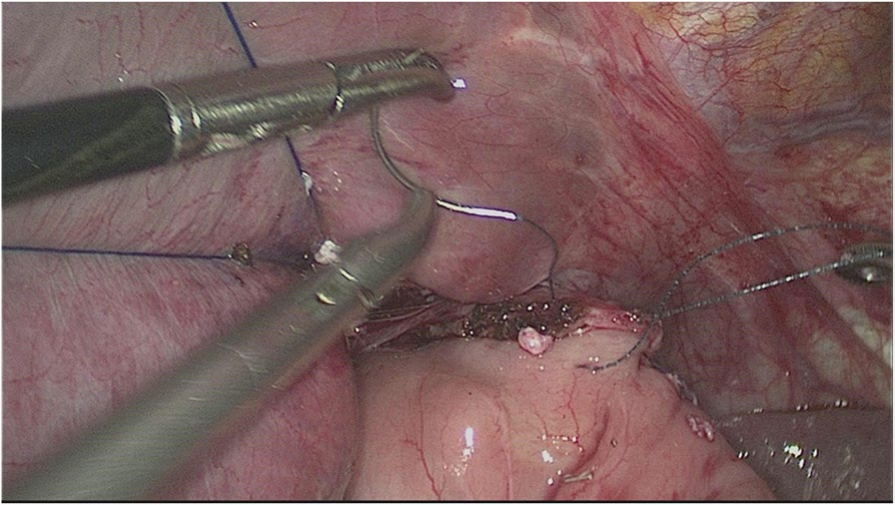
A barbed thread was used under laparoscopy to hand suture and close the joint opening

**Fig. 9:**
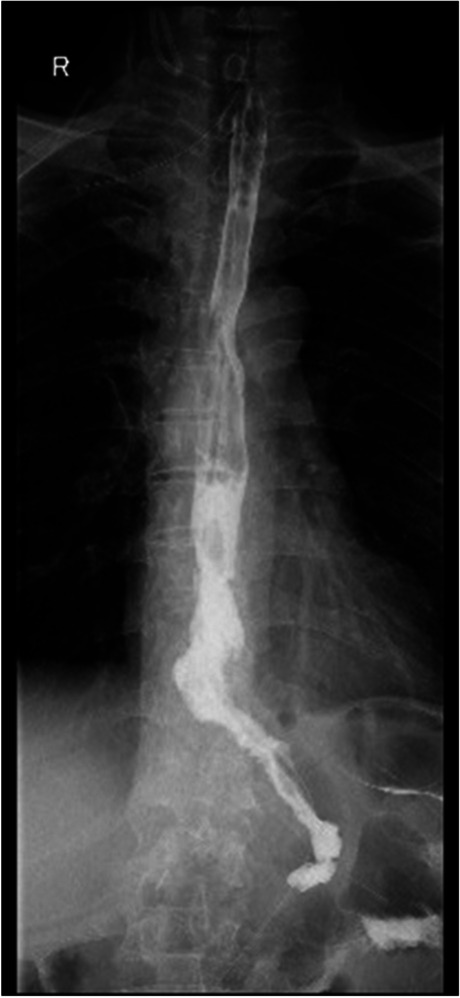
Postoperative angiographic examination showing a smooth anastomotic stoma

The esophagus was rotated 90° clockwise to make its cutting and closure lines in a sagittal position (Fig. 1). According to the operation space, it could be necessary to disconnect part of the diaphragm foot or free the left liver ligament. An ultrasonic knife or electric spatula was used to cut the closure line parallel to the esophagus on the right posterior wall of the esophagus’s broken end from the dorsal side to the ventral side to expose the mucosa (Fig. 2). An intraoperative gastric tube was used to guide the anastomotic channel (Fig. 3). The jejunum and its mesangium were cut 20 cm from the flexion ligament with a small incision or laparoscopy (Fig. 4). A small hole was made at the contralateral mesangial border 5 cm from the jejunum’s distal end, and a 45-mm linear stapler was inserted via the 12-mm trocar on the upper left side (Fig. 5). The distal jejunum was lifted through the anterior or posterior transverse colon until reaching the lower esophagus. A 45-mm linear stapler was inserted via the 12-mm trocar on the upper or lower left side to bring the right posterior wall of the esophagus and the jejunum to the mesangium border, forming a structure with the esophagus on the upper left and the jejunum on the lower right. The arms of the linear stapler were put into the opening of the esophagus and the jejunum. It was inserted to the stapling surface through the jejunum opening and the non-stapling surface through the esophagus opening as guided by the stomach tube (Fig. 6). The esophagus-jejunum side-to-side anastomosis was performed (Fig. 7). During anastomosis, the direction was adjusted so as not to damage the mesenteric blood vessels. A thorough check was conducted to confirm a complete anastomosis line, without bleeding, perforation, and false channel. Then, a barbed thread was used under laparoscopy to hand suture and close the joint opening (Fig. 8). Jejunum side-to-side anastomosis could be done under laparoscopy or through a small incision as needed. The seromuscular layer of each anastomotic stoma was strengthened with sutures, the Peterson hiatus was closed, and the duodenal stump was embedded. A 3–5-cm auxiliary incision was made under the xiphoid process or midway above the umbilical hole, and the specimen was taken out after a protector was used to protect the incision. A jejunal nutrition tube was routinely inserted, and the drainage tube was placed according to the intraoperative conditions. The postoperative angiographic examination showed a smooth anastomotic stoma (Fig. 9).

#### Digestive tract reconstruction using the conventional assisted method

An 8–10-cm auxiliary incision was made under the xiphoid process and in the middle of the upper abdominal area. After a protector was used to protect the incision, purse-string pliers were used to clamp and disconnect the esophagus under direct vision. The anvil head of the circular stapler was placed at the broken end of the esophagus. The purse suture was tightened into the anastomosis end of the esophagus. The mesangial vessels of the jejunum were separated and ligated 20 cm away from the ligament of flexion, the jejunum was cut off, and anterior colonic anastomosis was performed. The main body of the circular stapler was inserted deep via the opening of the distal jejunum, and the center rod was extended to the mesangial limbus. The anvil head at the anastomosis end of the esophagus was connected to the center rod at the anastomosis end of the jejunum to complete the anastomosis on the esophagus-jejunum side. It was tightened securely, and the distal stump of the jejunum was cut, and the stapler was closed. The mesangial direction and the blood supply of the anastomosis were carefully confirmed. The jejunum side-to-side anastomosis was performed. The seromuscular layer of each anastomotic stoma was strengthened with sutures, the Peterson hiatus was sutured, and the duodenal stump was embedded. A jejunum feeding tube was routinely placed, and the drainage tube was placed according to the intraoperative conditions.

#### Data collection

The observational indicators were 1) intraoperative conditions (operation time, esophagus-jejunum anastomosis time, intraoperative blood loss, number of lymph node dissected, length of the upper resection margin, and length of the auxiliary incision), 2) postoperative recovery (postoperative pain, the time of first postoperative anal exhaust, the time of first postoperative liquid food intake, the time of postoperative stay and postoperative early complications), and 3) postoperative complications (postoperative early complications and postoperative late complications), 4) quality of life before and after the operation. The tumor TNM staging was based on the 8th edition of the American Joint Committee on Cancer (AJCC) [18]. The postoperative pain degree was graded by oral description rating [19]. Quality of life assessment: The Chinese version of the Quality of Life Questionnaire Core 30 (QLQ-C30) designed by the European Organisation for Research and Treatment of Cancer (EORTC) and EORTC Quality of Life Questionnaire-Stomach (QLQ-STO22) were used to assess patients’ quality of life [20, 21]. The higher the scores of items in the function domain and better overall health conditions, the better the quality of life; further, the higher the scores of items in the symptom domain, the poorer the quality of life.

#### Follow-up

Follow-up was conducted routinely by outpatient visits or by telephone interview every 3 months for the first 2 years, then once every 6 months. The follow-up ended on March 31, 2023.

### Statistical analysis

SPSS 22.0 (IBM, Armonk, NY, USA) was used for analysis. Measurement data with skewed distribution were represented as M (range), and Mann–Whitney U rank.

sum test was used for comparison between groups. Measurement data with normal distribution were represented as means ± standard deviations, and t test was used for comparison between groups. Categorical data are expressed as n (%) and were compared using the chi-square test or Fisher’s exact test. *P*-values < 0.05 were considered statistically significant.

## Results

### Characteristics of the patients

The clinicopathological data of 242 patients with gastric cancer were collected. There were 164 males and 78 females, with a median age of 62.868 ± 10.600 years. Among them, 78 patients underwent a totally laparoscopic total gastrectomy with a linear stapler using the modified overlap method, and 164 underwent laparoscopic-assisted total gastrectomy with a circular stapler (conventional assisted group). There were no differences between the two groups regarding age, sex, body mass index (BMI), preoperative albumin, ASA grade, tumor site, maximum tumor diameter, histological grade, T stage and N stage (all *P* > 0.05) (Table 1).

**Table 1:** Clinicopathological characteristics

	Modified overlap anastomosis	Conventional incision-assisted anastomosis	*P* value
Number	78	164	
Sex			0.153
Male	48	116	
Femal	30	48	
Age (x ± s, years)	65 (28–83)	63 (27–83)	0.052
BMI (M (range), kg/m^2^)	22.082 (14.447–29.745)	22.020 (14.191–35.853)	0.820
Preoperative albumin (x ± s, g)	39.336 ± 4.320	38.479 ± 5.849	0.202
Maximum tumor diameter (M (range),, cm)	4 (0.9–11)	4.589 (0.5–9)	0.051
Histological grade			0.311
Poor	35	87	
Moderately	34	66	
Well	9	11	
Tumor stage			
T stage			0.106
T1	11	13	
T2	12	13	
T3	31	77	
T4	24	61	
N stage			0.743
N0	30	53	
N1	11	21	
N2	13	30	
N3	24	60	

### Intraoperative conditions

In the modified overlap group, the operation time was 223.45 (170.2–253.5) min, the esophagus-jejunum anastomosis time was 29.9 (24.4–36.4) min, the intraoperative blood loss was 53 (17–87) ml, the number of lymph nodes dissected was 37.923 ± 11.228, the length of the upper incision margin was 2 (1.3–4) cm, and the length of the auxiliary incision was 5 (4.3–5.9) cm. In the conventional assisted group, the corresponding values were 218.9 (132.5–387.5) min, 29.4 (24.7–36.2) min, 50.5 (17–81) ml, 34.963 ± 11.193, 2 (1.2–4.5) cm, and 8.2 (7.3–9.1) cm, respectively. There was a significant difference in the length of the auxiliary incision between the two groups (*P* < 0.05), but the other variables were not different between the two groups (all *P* > 0.05) (Table 2).

**Table 2:** Operation conditions

	Modified overlap anastomosis	Conventional incision-assisted anastomosis	*P* value
The operation time (M (range), min)	223.45 (170.2–253.5)	218.9 (132.5–387.5)	0.370
The esophagus-jejunum anastomosis time (M (range), min)	29.9 (24.4–36.4)	29.4 (24.7–36.2)	0.276
The intraoperative blood loss (M (range), ml)	53 (17–87)	50.5 (17–81)	0.780
the number of lymph nodes dissected (x ± s)	37.923 ± 11.228	34.963 ± 11.193	0.056
the length of the upper incision margin (M (range), cm)	2 (1.3–4)	2 (1.2–4.5)	0.061
the length of the auxiliary incision (M (range), cm)	5 (4.3–5.9)	8.2 (7.3–9.1)	0.000

### Postoperative recovery

Compared with the conventional assisted group, the modified overlap group showed milder postoperative pain (mild pain, 80.8% vs. 61.6%, *P* < 0.05), shorter time of first postoperative anal exhaust (3 (1–6) vs. 3 (1–8) days, *P* < 0.05), shorter time of first postoperative liquid food intake (4 (1–10) vs. 6 (4–13) days, *P* < 0.05), and shorter days of postoperative stay (9 (6–16) vs. 12 (8–21) days, *P* < 0.05) (Table 3).

**Table 3:** Postoperative recovery

	Modified overlap anastomosis	Conventional incision-assisted anastomosis	*P* value
Postoperative pain			0.009
Mild	63	101	
Moderate	13	56	
Severe	2	7	
The time of first postoperative anal exhaust (M (range), d)	3 (1–6)	3 (1–8)	0.003
The time of first postoperative fluid intake (M (range), d)	4 (1–10)	6 (4–13)	0.000
The time of postoperative stay (M (range), d)	9 (6–16)	12 (8–21)	0.000

### Postoperative complications

Postoperative early and late complications were showed in Table 4. All complications were improved by conservative treatment. There were no differences between the two groups regarding postoperative early and late complications (all *P* > 0.05).

**Table 4:** Postoperative complications

	Modified overlap anastomosis	Conventional incision-assisted anastomosis
Postoperative early complications		0.459
Anastomotic fistula	0	1
Anastomotic hemorrhage	0	1
Anastomotic stricture	0	1
Intestinal obstruction	1	2
Pulmonary infection	1	1
Incision infection	0	1
Others	0	0
Postoperative late complications		0.556
Anastomotic stricture	0	1
Intestinal obstruction	1	1
Reflux esophagitis	1	1
Dumping syndrome	1	1
Others	0	0

### Quality of life before and after the operation

A comparison of the scores of QLQ-C30 and QLQ-STO22 items in the two pre-operative groups revealed no statistically significant differences (both groups, *P* > 0.05). The QLQ-C30 scale three years after the operation showed low scores in function and symptom domains in the improved overlap and conventionally assisted groups, with no statistically significant differences (*P* > 0.05). The scores of the QLQ-STO22 scale 3 years after the operation showed significantly lower scores for difficulty swallowing and eating restriction symptoms in the improved overlap group than those in the conventionally assisted group (*P* < 0.05). However, the differences in scores of other symptoms between the two groups were not statistically significant (*P* > 0.05) (Tables 5 and 6).

**Table 5:** QLQ-C30

	Modified overlap anastomosis	Conventional incision-assisted anastomosis	*P* value
General health status			
Preoperative	62.231 ± 17.499	63.482 ± 19.617	0.632
3 years after operation	64.333 ± 20.721	62.024 ± 20.526	0.416
Functional domain			
Physical function			
Preoperative	85.385 ± 15.214	84.884 ± 13.750	0.798
3 years after operation	88.154 ± 16.323	83.713 ± 18.313	0.069
Role function			
Preoperative	64.833 ± 16.898	61.146 ± 17.833	0.128
3 years after operation	63.821 ± 15.556	62.932 ± 16.933	0.696
Emotional function			
Preoperative	76.167 ± 14.082	72.329 ± 16.296	0.075
3 years after operation	72.321 ± 16.373	75.183 ± 15.794	0.194
Cognitive function			
Preoperative	87.167 ± 20.505	86.476 ± 20.378	0.806
3 years after operation	83.269 ± 18.384	84.165 ± 17.268	0.712
Social function			
Preoperative	57.910 ± 13.262	54.909 ± 13.836	0.111
3 years after operation	60.641 ± 16.359	58.116 ± 13.382	0.204
Symptom domain			
Fatigue			
Preoperative	31.282 ± 14.549	33.445 ± 12.876	0.243
3 years after operation	34.513 ± 17.430	35.713 ± 14.608	0.576
Nausea and vomiting			
Preoperative	22.782 ± 12.709	24.268 ± 12.103	0.381
3 years after operation	22.205 ± 9.043	23.585 ± 11.149	0.341
Pain			
Preoperative	14.397 ± 7.477	16.329 ± 8.962	0.100
3 years after operation	11.744 ± 7.649	12.610 ± 6.922	0.380
Dyspnea			
Preoperative	9.821 ± 5.214	9.433 ± 5.178	0.588
3 years after operation	9.692 ± 4.724	10.012 ± 4.777	0.626
Insomnia			
Preoperative	25.936 ± 11.700	26.305 ± 10.233	0.803
3 years after operation	19.782 ± 9.082	21.457 ± 9.266	0.187
Appetite loss			
Preoperative	17.602 ± 9.215	17.738 ± 10.304	0.922
3 years after operation	17.897 ± 9.353	16.396 ± 9.198	0.239
Constipation			
Preoperative	17.103 ± 9.191	16.811 ± 8.820	0.813
3 years after operation	18.090 ± 9.274	18.939 ± 8.713	0.488
Diarrhea			
Preoperative	14.462 ± 7.584	15.189 ± 7.853	0.497
3 years after operation	15.539 ± 9.025	16.195 ± 8.358	0.578
Financial difficulties			
Preoperative	50.795 ± 18.279	48.585 ± 18.341	0.381
3 years after operation	44.487 ± 16.790	48.537 ± 16.516	0.077

**Table 6:** QLQ-STO22

	Modified overlap anastomosis	Conventional incision-assisted anastomosis	*P* value
Dysphagia			
Preoperative	16.821 ± 10.766	15.781 ± 10.089	0.464
3 years after operation	16.449 ± 10.688	21.152 ± 11.924	0.003
Pain			
Preoperative	19.077 ± 10.693	21.555 ± 12.577	0.135
3 years after operation	15.718 ± 10.966	18.616 ± 10.785	0.053
Reflux			
Preoperative	31.154 ± 14.389	29.945 ± 13.181	0.518
3 years after operation	19.115 ± 12.645	21.451 ± 11.383	0.151
Feeding limit			
Preoperative	18.577 ± 8.558	20.415 ± 8.728	0.125
3 years after operation	13.936 ± 7.400	16.866 ± 9.001	0.013
Anxiety			
Preoperative	34.013 ± 15.042	37.646 ± 17.300	0.113
3 years after operation	33.718 ± 14.269	33.335 ± 16.346	0.860
Dry mouth			
Preoperative	27.885 ± 12.526	28.067 ± 13.186	0.919
3 years after operation	29.833 ± 12.147	28.116 ± 13.453	0.340
Taste change			
Preoperative	15.205 ± 8.904	14.012 ± 8.491	0.316
3 years after operation	13.308 ± 7.508	14.701 ± 8.534	0.219
Behavior			
Preoperative	25.436 ± 13.333	26.384 ± 11.268	0.588
3 years after operation	23.885 ± 12.434	22.421 ± 10.537	0.342
Hair loss			
Preoperative	19.064 ± 9.523	20.366 ± 10.017	0.338
3 years after operation	19.013 ± 10.046	20.165 ± 10.529	0.420

### Follow-up

Out of the 242 patients, 237 were followed. Among the 78 patients in the modified overlap group, 76 were followed for a median of 53 (36–75) months. Among the 164 patients in the conventional assisted group, 161 were followed for a median of 67.0 (45.0–87.0) months. There was no recurrence among the patients in the modified overlap group. There was one patient with tumor recurrence and liver metastasis in the conventional assisted group. There was no death.

## Discussion

Inaba et al. [16] proposed the totally laparoscopic overlap method, and a modified overlap method is used at the authors’ hospital. This study aimed to compare the postoperative characteristics and anastomosis complications of a modified overlap anastomosis and conventional incision-assisted anastomosis for laparoscopic total gastrectomy. The results suggest that compared with traditional auxiliary incision anastomosis, patients undergoing totally laparoscopic total gastrectomy with modified overlap anastomosis have a smaller incision and a better postoperative recovery.

At present, the common way to reconstruct the digestive tract in laparoscopic surgery for gastric cancer is Roux-en-Y anastomosis, but the esophagus-jejunum anastomosis remains controversial [22–31]. The overlap method proposed by Inaba et al. [16] allows an operation in a more limited space while achieving a higher anastomosis position and thus a better anastomotic stoma path. Moreover, after surgery, the esophagus and the jejunum are peristaltic and conform to the digestive tract’s normal physiological functions. Its excellent effect has been affirmed by many studies [32–36].

Some authors have suggested partial modifications of the original overlap method, including the posterior disjunction method proposed by Huang et al. [37], the inverted T anastomosis by Nagai et al. [38], and the improvements made by Lee et al. [39], all of which result in satisfactory therapeutic effects. The authors of this study also attempted to improve the overlap method and showed that compared with the conventional incision-assisted method, the modified overlap method could effectively ensure negative margins without prolonging the operation time or increasing intraoperative blood loss and tumor recurrence.

After a laparoscopic total gastrectomy, the incidence of esophagus-jejunum anastomotic stoma fistula is 3.0%-6.5%, that of anastomotic stoma hemorrhage is 1.8%-4.0%, and that of anastomotic stoma stenosis is 3.2%-17.0% [38–48]. This study showed that the above early complications in the modified overlap group were lower than in the previous studies and that there were no statistically significant differences with the conventional assisted group. In addition, compared with the conventional assisted group, the modified overlap group showed smaller auxiliary incision, milder postoperative pain, and shorter first postoperative anal exhaust time, first postoperative liquid food intake time, and postoperative stay.

Regarding late complications, the number of incidences of post-operative anastomotic stricture in the conventionally assisted group was more than in the improved overlap group; however, the difference was not statistically significant. Theoretically, the high incidence rate of circular anastomotic stenosis can be mainly attributed to the fact that the placement of the anvil of the circular stapler was highly restricted by the oesophageal diameter. An excessively larger diameter may lead to oesophageal mucosal damage, while an excessively small diameter may lead to anastomotic stenosis. Additionally, the anastomotic stoma is perpendicular to the oesophageal lumen, which easily causes scar contracture after recovery, inducing an intense sense of choking when swallowing and, in worst cases, difficulty swallowing. The linear cutter stapler has a staple cartridge with a larger diameter. The anastomotic stoma is parallel to the oesophageal direction, making it easier to avoid the said problems and resulting in a lower risk of post-operative anastomotic stricture. However, the incidence rates of anastomotic stoma-related complications did not exhibit any statistically significant difference in the case of the two groups considered in this research, which is consistent with the results of other studies.

We assessed patients’ quality of life using a combination of EORTC QLQ-C30 and EORTC QLQ-STO22 to further explore the effects of these two surgical approaches on other aspects of quality of life, such as post-operative physical function, gastrointestinal symptoms and economic conditions. The comparison of quality of life indicators from QLQ-C30 and QLQ-STO22 3 years after the operation showed that the modified overlap group had lower scores in dysphagia and feeding limit, which was attributed to the wider anastomotic stoma made by the linear cutter stapler with milder associated complications. Generally, anastomosis using a linear cutter stapler requires more staple cartridges, which are more expensive than circular stapler cartridges, resulting in higher surgical consumable costs and heavier financial burdens on patients. However, 3 years after surgery is relatively long that there is no significant difference in financial burdens between the two groups. The two groups showed no statistically significant differences in other aspects of quality of life, such as reflux symptoms, general health status and physical function, indicating that applying a linear cutter stapler to laparoscopic esophagojejunostomy is advantageous in relieving post-operative dysphagia and feeding limit. The conventional assisted group showed no significant difference in other aspects of quality of life. Further studies are required to explore its long-term effect on the quality of life.

The technical points of this modified overlap method should be examined. In the original overlap method, the opening of the lower stump of the esophagus is on the left side and is often blocked by the diaphragm. Since the esophagus tends to retract into the mediastinum after being cut, excessive traction during the operation will easily cause tears in the esophagus muscle layer, thereby increasing the risks of postoperative anastomotic stoma fistula and hemorrhage. In the modified method, the esophagus is rotated 90° clockwise to make the shared opening more in the front, creating a better surgical field of vision. After the esophagus was rotated, the esophagus is opened on the right back wall. At this point, the linear stapler and the esophagus’s longitudinal axis are at a certain angle, which could avoid damage to the esophagus.

In the conventional method, the chief surgeon stands on the right side, and the linear stapler is inserted via the bottom right trocar. The operation is often far from the esophagus-jejunum anastomosis. At this point, the trocar can easily move up and inward. In the modified method, the chief surgeon stood on the patient’s left side, and a 45-mm linear stapler was placed via the upper left trocar for esophagus-jejunum anastomosis. The left position is more convenient than the right for the chief surgeon because the linear stapler would be placed on the left side and closer to the anastomosis site. At this point, the esophagus and the jejunum are aligned axially, which allows the adjustment of angle and force. The obstruction of the liver is also reduced to provide a better view and space.

The esophagus-jejunum anastomosis is the most critical step in the overlap method. In this study, a 45-mm linear stapler was routinely used, which delivers a large diameter of an anastomotic stoma that allows flexible operations within a limited space. During anastomosis, the esophagus was on the upper left and the jejunum on the lower left, which conformed to the normal spatial course and the operating preferences of the chief surgeon on the left side. The esophagus-jejunum anastomosis can be divided into anterior colonic and posterior colonic anastomosis according to the jejunum’s position relative to the transverse colon. Compared with anterior colonic anastomosis, the posterior colonic anastomosis can shorten the esophagus-jejunum anastomosis’s distance and reduce the tension of the anastomotic stoma. The lifted jejunum is located behind the colon, bringing less compression onto the transverse colon than the anterior colonic one. Moreover, since it is not easy to adhere to the abdominal wall, the probability of intestinal obstruction after the operation is low. Meanwhile, after anastomosis, the digestive tract conforms to the normal physiological structure, which is conducive to the digestive functions’ rapid recovery. For this reason, posterior colonic anastomosis was routinely used in this study. As for the height of the esophagus’s stump, to avoid diaphragm obstruction during high anastomosis, the diaphragm hiatus was entered. If necessary, part of the diaphragm foot can be cut to expand the space and reduce the operating tension. It should be noted that the match between the opening edges of the esophagus and the jejunum should be ensured, which could reduce the tension of the anastomotic stoma.

After the esophagus-jejunum side-to-side anastomosis, inserting a gastric tube in advance can confirm whether the anastomotic stoma is unobstructed, whether there are stenosis and the formation of false channels. The shared opening can be sutured intermittently or continuously according to the experience or preferences of the surgeon. It is also one of the technical difficulties of the original overlap method and requires a high laparoscopic suture level. In the modified method, continuous suture with a barbed thread was used, which offered simple operations and accurate effect. If an anastomosis stapler were used to close the shared opening, there would be risks of high space limitation and anastomotic stoma stenosis. In addition, due to the thick seromuscular layer and thin mucosa of the jejunum, double-layer sutures were routinely performed, which could better ensure the quality of anatomic stoma.

Of course, this study has limitations. The sample size was small since the patients were from a single hospital and operated by a single team of surgeons. Because of the study’s retrospective nature, only the data that were in the charts could be analyzed. Thus, large-scale and multicenter prospective studies should be conducted in the future.

In conclusion, the modified overlap anastomosis is associated with better minimal invasiveness and faster post-operative recovery than the conventionally assisted incision anastomosis, along with an almost equal incidence rate of postoperative complications and capability to reduce post-operative symptoms such as difficulty swallowing and eating restrictions. Therefore, it is a safe and effective digestive tract reconstruction approach.

## Data Availability

The data sets used and analyzed during the current study are available from the corresponding author on reasonable request.
